# Supplementary figures and images for: Distribution of nematophagous fungi and soil-transmitted helminths in outdoor built environments across Latin America
Source: PLoS Negl Trop Dis. 2026 Feb 17;20(2):e0013990. doi: 10.1371/journal.pntd.0013990 (PMC12923129; doi:10.1371/journal.pntd.0013990)

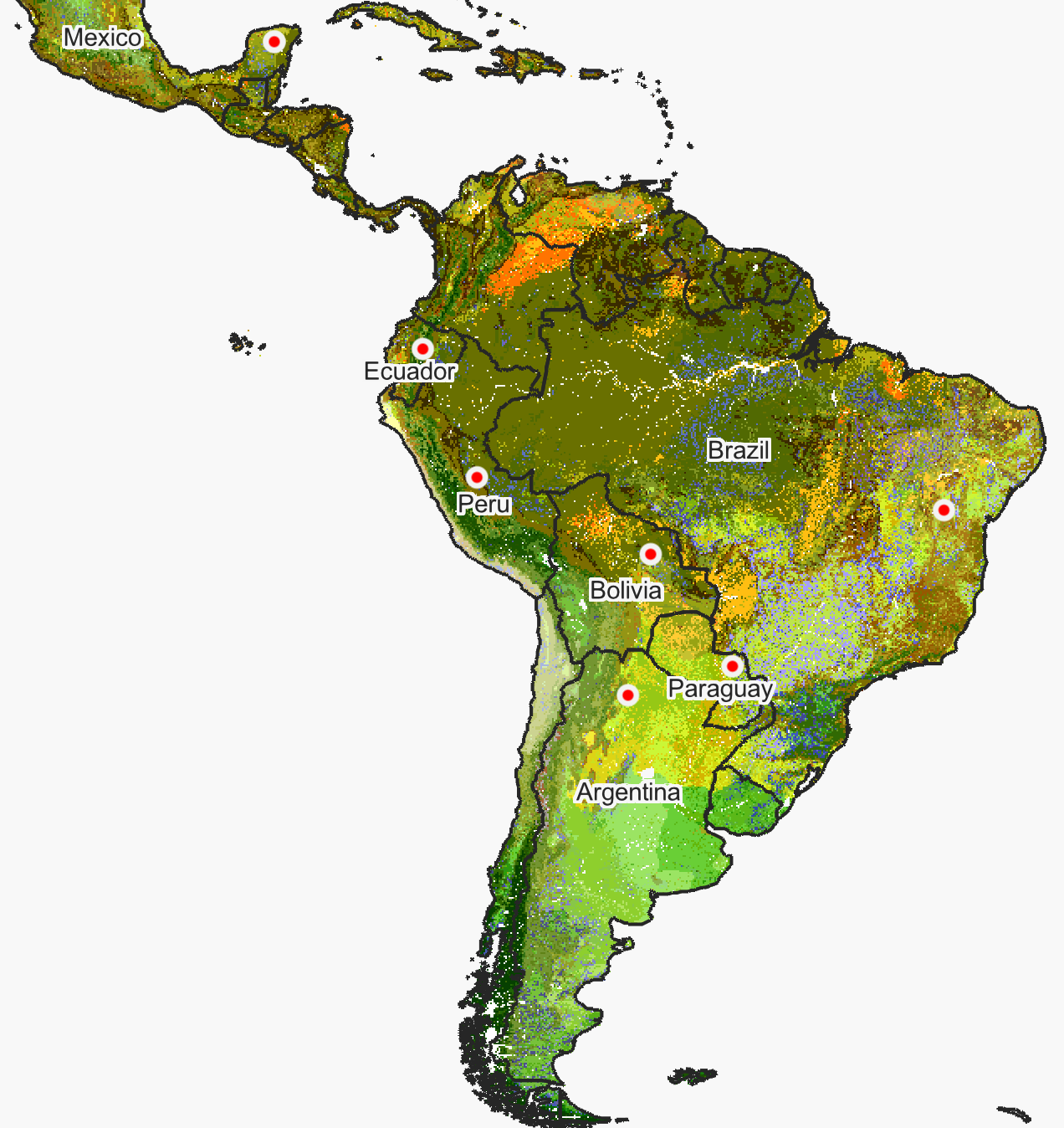

Supplement: S1 Fig — Details are in Table 1 (World Terrestrial Ecosystems retrieved December 8, 2025 using ArcGIS Online by Environmental Systems Research Institute, https://www.arcgis.com/apps/mapviewer/index.html?layers=926a206393ec40a590d8caf29ae9a93e). (TIFF) [file pntd.0013990.s001.tiff]

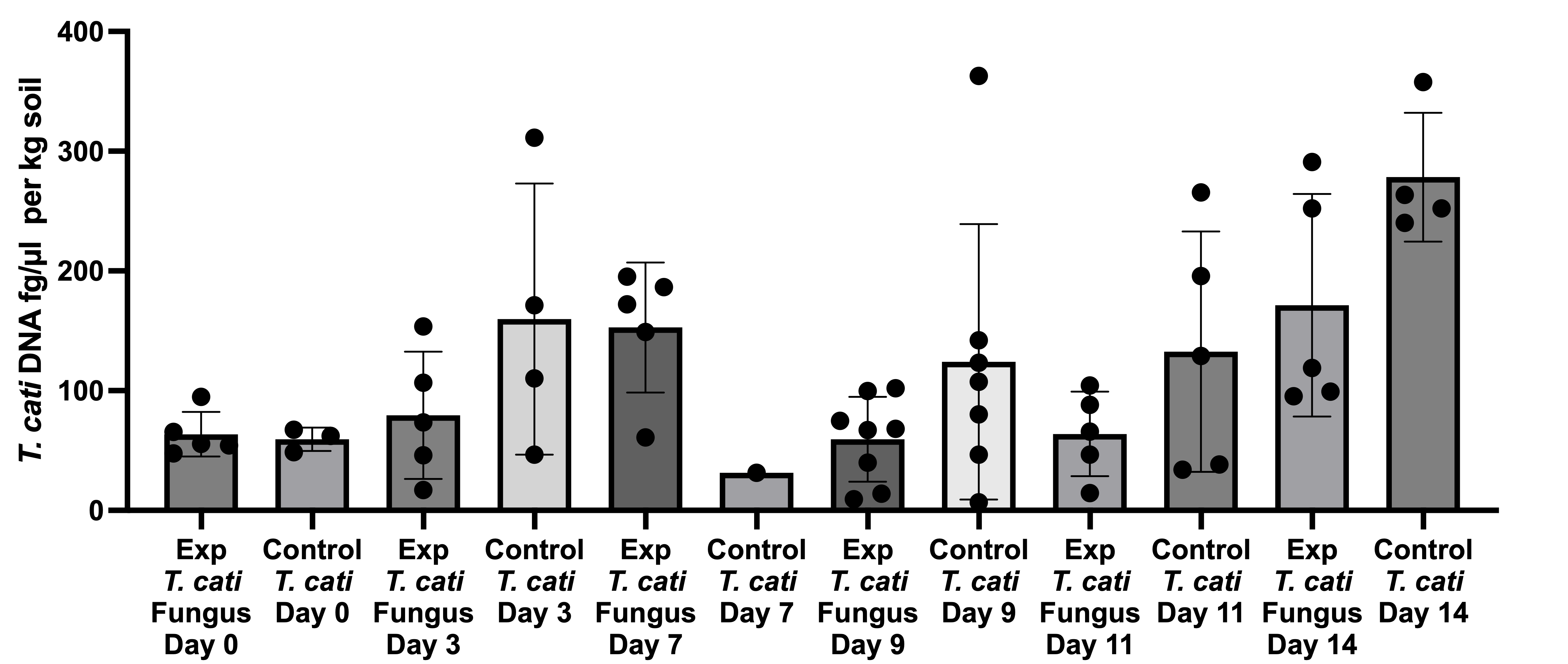

Supplement: S2 Fig — (TIFF) [file pntd.0013990.s004.tiff]

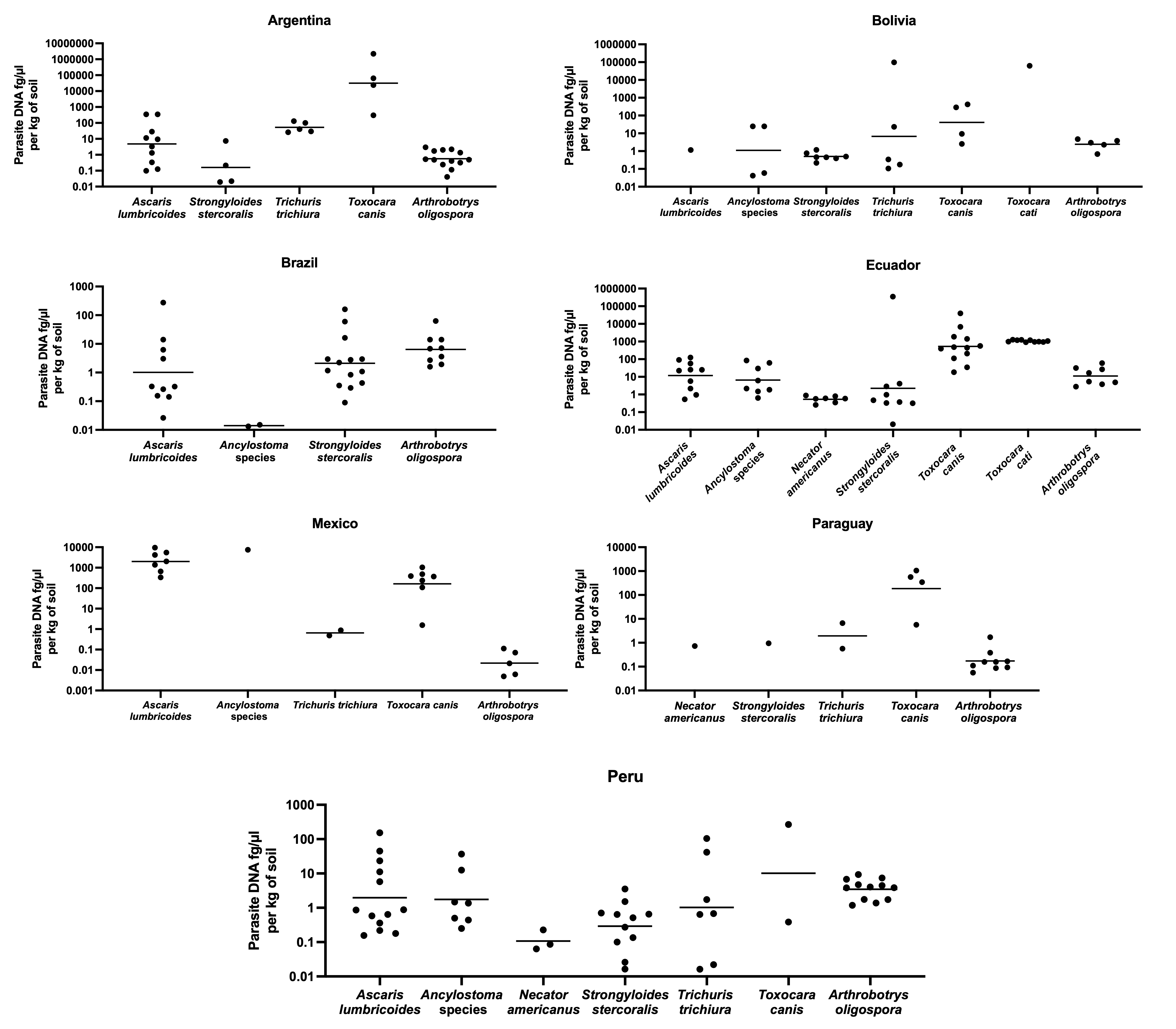

Supplement: S3 Fig — Values in Table 2. (TIFF) [file pntd.0013990.s005.tiff]
