# Supplementary material for: Distribution of nematophagous fungi and soil-transmitted helminths in outdoor built environments across Latin America
Source: PLoS Negl Trop Dis. 2026 Feb 17;20(2):e0013990. doi: 10.1371/journal.pntd.0013990 (PMC12923129; doi:10.1371/journal.pntd.0013990)
Supplement: S1 Table — (DOCX) [file pntd.0013990.s002.docx]

| **Environmental Sampling** | **qPCR** |
| --- | --- |
| Up to 50 grams | Target genes (supplemental table 2) |
| Argentina, Bolivia, Brazil, Ecuador, Mexico, Paraguay, Peru | Hold stage 95°C, 20 sec; Amplification Denaturation 95°C, 1 sec; Annealing 60°C, 20 sec. |
| Table 1 | 2x TaqMan® Fast Advanced Master Mix (Applied Biosystems, Foster City, CA) 3.5 µl |
| Stored at 4°C and DNA extracted within 1 month | 2 µl of template |
| Exogenous DNA was used as an internal control to validate the extraction method. All samples had the internal control detect via qPCR | Primers were used at 900 nM (Thermofisher)  Probe was used at 100 nM (Thermofisher) |
| **Sample Treatment** | QS7 Pro Fast Real-time PCR System (Applied Biosystems, Waltham, Massachusetts, USA)  Or ABI 7500 Real-time PCR System (Applied Biosystems)  Or Chia Portable Real-time PCR (Chia Bio, Santa Clara, CA) |
| Soil washed with PBS and 0.05% Tween 20 | 2 µl of PCR-water was used as a negative control |
| Flotation with 35.6% Sodium Nitrate solution or sugar solution (Specific Gravity 1.3) | Plasmids containing target parasite gene sequences was used as positive control |
| **Sample Reduction** | An exogenous DNA internal control was tested and all samples tested positive for the internal control Ct median  33.80 (31.94 - 37.08). |
| Samples are concentrated by a factor of 500 | **Analysis - qPCR** |
| **Nucleic Acid Extraction** | Positive control standard curves were performed in duplicate |
| MP fastDNA Spin kits for soil | Samples were tested in single |
| DNA eluent is 100 µl and stored at -20°C | All positive controls were compared to a set of known Ct values and were all within 5% range |
|  | Lowest standard measured was approximately 0.1 fg/µl per kg of soil |
|  | Automatic baseline and a threshold of 0.40 was used for all parasites |
