# Supplementary material for: Distribution of nematophagous fungi and soil-transmitted helminths in outdoor built environments across Latin America
Source: PLoS Negl Trop Dis. 2026 Feb 17;20(2):e0013990. doi: 10.1371/journal.pntd.0013990 (PMC12923129; doi:10.1371/journal.pntd.0013990)
Supplement: S2 Table — (DOCX) [file pntd.0013990.s003.docx]

| Helminth | Target region | Forward primer sequence (5’ to 3’)  Reverse primer sequence (5’ to 3’)  Probe sequence (5’FAM to 3’) |
| --- | --- | --- |
| *Ancylostoma* species | ITS-2 | GAATGACAGCAAACTCGTTGTTG ATACTAGCCACTGCCGAAACGT ATCGTTTACCGACTTTAG |
| *Ascaris lumbricoides* | ITS-1 | TGCACATAAGTACTATTTGCGCGTAT  CCGCCGACTGCTATTACATCA  GAGCCACATAGTAAATT |
| *Necator americanus* | ITS-2 | CTGTTTGTCGAACGGTACTTGC  ATAACAGCGTGCACATGTTGC  CTGTACTACGCATTGTATAC |
| *Strongyloides stercoralis* | 18s rRNA | GAATTCCAAGTAAACGTAAGTCATTAGC  TGCCTCTGGATATTGCTCAGTTC  ACACACCGGCCGTCGCTGC |
| *Toxocara canis* | ITS-2 | GCGCCAATTTATGGAATGTGAT  GAGCAAACGACAGCSATTTCTT  CCATTACCACACCAGCATAGCTCACCGA |
| *Toxocara cati* | ITS-2 | ACGCGTACGTATGGAATGTGCT  GAGCAAACGACAGCSATTTCTT  TCTTTCGCAACGTGCATTCGGTGA |
| *Trichuris trichiura* | ITS-1 | TCCGAACGGCGGATCA  CTCGAGTGTCACGTCGTCCTT  TTGGCTCGTAGGTCGTT |
| *Internal Amplification Control* (IAC) | Synthetic sequence | CTAACCTTCGTGATGAGCAATCG  GATCAGCTACGTGAGGTCCTAC  TCGATGCACTCCAGTCCTCCT |
| *ITS = internal transcribed spacer; rRNA = ribosomal RNA | |  |
