# Supplementary material for: Distribution of nematophagous fungi and soil-transmitted helminths in outdoor built environments across Latin America
Source: PLoS Negl Trop Dis. 2026 Feb 17;20(2):e0013990. doi: 10.1371/journal.pntd.0013990 (PMC12923129; doi:10.1371/journal.pntd.0013990)
Supplement: S3 Table — (DOCX) [file pntd.0013990.s006.docx]

| **Argentina** | | | | |
| --- | --- | --- | --- | --- |
| **Helminth** | **ODD RATIO** | **95% CI Lower** | **95% CI Upper** | **P value** |
| *Ascaris lumbricoides* | 1.2 | 0.20 | 14.33 | >0.99 |
| *Strongyloides stercoralis* | 0.38 | 0.053 | 5.3 | 0.40 |
| *Trichuris trichiura* | 0.032 | 0.0026 | 0.23 | 0.0014* |
| *Toxocara canis* | Infinity | 0.16 | Infinity | >0.99 |
| Any helminth | 0.60 | 0.17 | 2.2 | 0.44 |
| **Bolivia** | | | | |
| *Ascaris lumbricoides* | Infinity | 0.0063 | Infinity | >0.99 |
| *Ancylostoma* species | Infinity | 0.044 | Infinity | >0.99 |
| *Strongyloides stercoralis* | Infinity | 0.096 | Infinity | >0.99 |
| *Trichuris trichiura* | Infinity | 0.058 | Infinity | >0.99 |
| *Toxocara* species | 0.19 | 0.017 | 2.87 | 0.25 |
| Any helminth | 1.1 | 0.16 | 14.12 | >0.99 |
| **Brazil** | | | | |
| *Ascaris lumbricoides* | 0.24 | 0.038 | 1.42 | 0.15 |
| *Ancylostoma* species | Infinity | 0.044 | Infinity | 0.65 |
| *Strongyloides stercoralis* | Infinity | 24.94 | Infinity | <0.0001 |
| Any helminth | 0.93 | 0.21 | 4.86 | >0.99 |
| **Ecuador** |  |  |  |  |
| *Ascaris lumbricoides* | 0.034 | 0.0080 | 0.21 | 0.0002 * |
| *Ancylostoma* species | 0.57 | 0.069 | 7.26 | 0.50 |
| *Necator americanus* | 0.48 | 0.053 | 6.3 | 0.45 |
| *Strongyloides stercoralis* | 0.057 | 0.013 | 0.26 | 0.0018 * |
| *Trichuris trichiura* | 0.23 | 0.032 | 3.4 | 0.29 |
| *Toxocara canis* | 0.094 | 0.025 | 0.39 | 0.0064 * |
| *Toxocara cati* | 0.28 | 0.051 | 1.58 | 0.18 |
| *Toxocara* species | 0.12 | 0.029 | 0.53 | 0.0072 * |
| Any helminth | 0.00 | 0.00 | 0.25 | 0.0003 * |
| **Mexico** | | | | |
| *Ascaris lumbricoides* | 0.11 | 0.020 | 0.78 | 0.063 |
| *Ancylostoma* species | Infinity | 0.0077 | Infinity | >0.99 |
| *Toxocara canis* | Infinity | 0.12 | Infinity | >0.99 |
| *Trichuris trichiura* | Infinity | 0.030 | Infinity | >0.99 |
| Any helminth | 0.30 | 0.057 | 1.87 | 0.22 |
| **Paraguay** | | | | |
| *Necator americanus* | 0.00 | 0.00 | 0.63 | 0.066 |
| *Strongyloides stercoralis* | 0.00 | 0.00 | 0.63 | 0.066 |
| *Toxocara canis* | Infinity | 0.059 | Infinity | >0.99 |
| *Trichuris trichiura* | Infinity | 0.031 | Infinity | >0.99 |
| Any helminth | 0.17 | 0.030 | 0.98 | 0.088 |
| **Peru** | | | | |
| *Ascaris lumbricoides* | 0.76 | 0.11 | 8.8 | 0.57 |
| *Ancylostoma species* | 0.37 | 0.049 | 4.6 | 0.36 |
| *Necator americanus* | Infinity | 0.054 | Infinity | >0.99 |
| *Strongyloides stercoralis* | 0.63 | 0.10 | 7.4 | 0.51 |
| *Trichuris trichiura* | Infinity | 0.031 | Infinity | >0.99 |
| *Toxocara canis* | 0.059 | 0.0031 | 1.23 | 0.12 |
| Any helminth | 0.67 | 0.19 | 2.4 | 0.70 |
