# Supplementary material for: Distribution of nematophagous fungi and soil-transmitted helminths in outdoor built environments across Latin America
Source: PLoS Negl Trop Dis. 2026 Feb 17;20(2):e0013990. doi: 10.1371/journal.pntd.0013990 (PMC12923129; doi:10.1371/journal.pntd.0013990)
Supplement: Attachment — Submitted filename: Response to Reviewers.docx [file pntd.0013990.s012.docx]

**Reviewers' Comments:**

Reviewer's Responses to Questions

**Key Review Criteria Required for Acceptance?**

As you describe the new analyses required for acceptance, please consider the following:

**Methods**

-Are the objectives of the study clearly articulated with a clear testable hypothesis stated?

-Is the study design appropriate to address the stated objectives?

-Is the population clearly described and appropriate for the hypothesis being tested?

-Is the sample size sufficient to ensure adequate power to address the hypothesis being tested?

-Were correct statistical analysis used to support conclusions?

-Are there concerns about ethical or regulatory requirements being met?

Reviewer #1: (No Response)

Reviewer #2: (No Response)

Reviewer #3: This study clearly states its objectives. The methods section is generally clear, but I have a couple of questions:
Please check that reference 24 is correct.
Could you please provide more details about the sampling sites, i.e., whether they were public areas such as parks or streets; and if there were any specific conditions that facilitated STH contamination?

*Reference 24 has been corrected.*

*This has been added for sampling site information.*

*All dirt samples were collected outside participants' houses, including the entrance, latrine, and patio, except for 45 samples from seven city parks in Huánuco, Peru (24). These locations are in resource-poor areas throughout Latin America, with outdoor latrines and limited access to proper sanitation. Latrines were limited-service latrines on the Joint Monitoring Programme (JMP) sanitation ladder, about 2 – 5 meters from the house’s entrance. Permission was obtained from tenants and local Public Health officials.*

**Results**


-Does the analysis presented match the analysis plan?

-Are the results clearly and completely presented?

-Are the figures (Tables, Images) of sufficient quality for clarity?

Reviewer #1: (No Response)

Reviewer #2: (No Response)

Reviewer #3: The results are clearly presented

**Conclusions**

-Are the conclusions supported by the data presented?

-Are the limitations of analysis clearly described?

-Do the authors discuss how these data can be helpful to advance our understanding of the topic under study?

-Is public health relevance addressed?

Reviewer #1: (No Response)

Reviewer #2: (No Response)

Reviewer #3: Although the statements on lines 400-406 are correct, please support them with ad hoc references.
The conclusion is appropriate.

*We have added 2 references.*

**Editorial and Data Presentation Modifications?**

Use this section for editorial suggestions as well as relatively minor modifications of existing data that would enhance clarity. If the only modifications needed are minor and/or editorial, you may wish to recommend “Minor Revision” or “Accept”.

Reviewer #1: (No Response)

Reviewer #2: (No Response)

Reviewer #3: This manuscript is interesting and presents new knowledge through a little-studied approach on the potential future use of Arthrobotrys oligospora for HTS control.

**Summary and General Comments**

Use this section to provide overall comments, discuss strengths/weaknesses of the study, novelty, significance, general execution and scholarship. You may also include additional comments for the author, including concerns about dual publication, research ethics, or publication ethics. If requesting major revision, please articulate the new experiments that are needed.

Reviewer #1: This manuscript studies the potential role of nematophagous fungi as an environmentally sustainable approach to reduce soil-transmitted helminths (STHs). The combination of laboratory-based experiments and multi-country environmental sampling is ambitious and, in principle, could provide valuable insights into parasite control at the soil level. However, in its current form, the study suffers from substantial conceptual, methodological, and interpretational limitations that critically undermine the validity of its conclusions. Here below are my main concerns:

The manuscript combines controlled in vitro experiments with a large observational soil survey; however, these two components are not mechanistically or causally integrated. The in vitro assays demonstrate fungal–nematode interactions under highly artificial conditions, whereas the environmental study relies solely on cross-sectional DNA detection. No evidence is provided that the mechanisms observed in vitro operate under natural soil conditions, rendering the linkage between the two datasets speculative.

*These 2 experiments are related in that both use the same parasite concentration, filtration, and DNA extraction. They use the same qPCR methods and complement each other by taking an in vitro controlled test and implementing into a real-world field study. However, we understand the reviewer’s concern. For this reason, we downplay the mechanistic interpretation between the two experiments throughout the paper, including the article title*

*We have labelled the in vitro as a controlled test and the field study as an observational cross-sectional study to not mislead the readers.*

Another major methodological flaw exists in the in vitro soil-based experiments. Commercial garden soil was used, and several control samples were found to naturally contain A. oligospora DNA. As a result, the control condition is not fungus-free, and qPCR cannot distinguish background fungal DNA from experimentally introduced fungus. This fundamental confounding undermines the validity of the reported reduction in Toxocara cati DNA.

*The use of commercial soil was on purpose to mimic the natural soil samples found in the Latin America observational study. The fact some control soils had fungal DNA shows how ubiquitous it is found. We have rerun the analysis omitting the control samples with no change in significance. We have also included this figure in the Supplemental section. This has been added to the results section “As a proof-of-principle test, control samples that contained A. oligospora DNA were removed from the in vitro analysis. The two-way ANOVA with log 10-transformed T. cati DNA remained significant on the impact of fungus on T. cati DNA (P = 0.0148) and no significance on time (P = 0.5318) or interaction (P = 0.3096)* ***(Supplemental Figure 2)****.”*

In the environmental study, the nematode qPCR assays are acknowledged to lack strict species specificity, yet the results are interpreted at the species level and extrapolated to human health relevance. In addition, only A. oligospora was assayed despite the known diversity of nematophagous fungi (e.g. Duddingtonia flagrans, Monacrosporium spp., Arthrobotrys spp.) in soil, making broad conclusions about fungal-mediated control unjustified.

*While strict species specificity is difficult by using molecular and also microscopy methods, this study uses a qPCR that maintains a high species sensitivity with no cross-reactivity between the helminths tested. The goals of this study did not focus on risk of human infection but the impact of A. oligospora on nematodes which include zoonotic helminths.*

*The other nematophagous fungi species were mentioned as background and not included in this pilot study.*

*The fungal-mediated control of helminths has been removed and now we focus on correlation of NF and helminths.*

Finally, the soil data are purely correlational, based on DNA detection that does not indicate organism viability or active killing, and no multivariable analyses were performed to account for major environmental confounders. Despite these limitations, the manuscript repeatedly implies active biological control of STHs in the environment, which is not supported by the presented data.

*We have now stated that this is a controlled laboratory and observational cross-sectional study.*

*We also mention that environmental factors will be used in future studies.*

The central conclusions regarding environmental control of soil-transmitted helminths by Arthrobotrys oligospora substantially exceed what can be supported by the current data. Addressing these limitations would require extensive redesign of both laboratory and field components of the study. Therefore, I do not consider the manuscript suitable for publication in its present form.

*We have reworded and restructured the claims of using a fungus for helminth control.*

*Including changing the title to “Distribution of Nematophagous Fungi and Soil-Transmitted Helminths in Outdoor Built Environments Across Latin America”*

Reviewer #2: (No Response)

Reviewer #3: This manuscript is well written and presents novel results. I suggest clarifying a few minor comments before publication.

PLOS authors have the option to publish the peer review history of their article ([what does this mean?](https://urldefense.proofpoint.com/v2/url?u=https-3A__track.editorialmanager.com_CL0_https-3A-252F-252Fjournals.plos.org-252Fplosntds-252Fs-252Feditorial-2Dand-2Dpeer-2Dreview-2Dprocess-2523loc-2Dpeer-2Dreview-2Dhistory_1_010f019bdb438beb-2D09f3354f-2Df6a1-2D4b93-2D9118-2De99d6e6bda93-2D000000_0CEaBl-2D0G9vQRRcXKCPp0KXNPTVsVPnvMU5XoLIApU8-3D246&d=DwMFaQ&c=ZQs-KZ8oxEw0p81sqgiaRA&r=ZEy0IyQ0Gydtbeyd2qTjxqzEHjVUKaDuw8GJEp5nVMA&m=JBdsibLhF6iqj8pZO8GChLamJcJa8DPfsg9uClHUntUHCnoOhfqV6iegZMxjFA2Q&s=Ni4Ii37HKXWZ04IeIbtdqvITS0hCPXhSvyGIALqFJWc&e=)). If published, this will include your full peer review and any attached files.

If you choose “no”, your identity will remain anonymous but your review may still be made public.

**Do you want your identity to be public for this peer review?** For information about this choice, including consent withdrawal, please see our [Privacy Policy](https://urldefense.proofpoint.com/v2/url?u=https-3A__track.editorialmanager.com_CL0_https-3A-252F-252Fwww.plos.org-252Fprivacy-2Dpolicy_1_010f019bdb438beb-2D09f3354f-2Df6a1-2D4b93-2D9118-2De99d6e6bda93-2D000000_-5FcgJbOsATDzoyDD5KMjrGEcan-2Dv-2D1qOx-2DOGvoGre7SM-3D246&d=DwMFaQ&c=ZQs-KZ8oxEw0p81sqgiaRA&r=ZEy0IyQ0Gydtbeyd2qTjxqzEHjVUKaDuw8GJEp5nVMA&m=JBdsibLhF6iqj8pZO8GChLamJcJa8DPfsg9uClHUntUHCnoOhfqV6iegZMxjFA2Q&s=paQIb1qU7QhKbTNrtfJq_kqq7lFqv6pzAZl92vtUPEI&e=).

Reviewer #1: No

Reviewer #2: No

Reviewer #3: No

**Figure resubmission:**

While revising your submission, we strongly recommend that you use PLOS’s NAAS tool (<https://ngplosjournals.pagemajik.ai/artanalysis>) to test your figure files. NAAS can convert your figure files to the TIFF file type and meet basic requirements (such as print size, resolution), or provide you with a report on issues that do not meet our requirements and that NAAS cannot fix.

After uploading your figures to PLOS’s NAAS tool - <https://ngplosjournals.pagemajik.ai/artanalysis>, NAAS will process the files provided and display the results in the "Uploaded Files" section of the page as the processing is complete. If the uploaded figures meet our requirements (or NAAS is able to fix the files to meet our requirements), the figure will be marked as "fixed" above. If NAAS is unable to fix the files, a red "failed" label will appear above. When NAAS has confirmed that the figure files meet our requirements, please download the file via the download option, and include these NAAS processed figure files when submitting your revised manuscript.

**Reproducibility:**

To enhance the reproducibility of your results, we recommend that authors of applicable studies deposit laboratory protocols in protocols.io, where a protocol can be assigned its own identifier (DOI) such that it can be cited independently in the future. Additionally, PLOS ONE offers an option to publish peer-reviewed clinical study protocols. Read more information on sharing protocols at <https://plos.org/protocols?utm_medium=editorial-email&utm_source=authorletters&utm_campaign=protocols>
